# Comparison of Various Criteria Determining the Direction of Crack Propagation Using the UDMGINI User Procedure Implemented in Abaqus

**DOI:** 10.3390/ma14123382

**Published:** 2021-06-18

**Authors:** Jakub Gontarz, Jerzy Podgórski

**Affiliations:** Department of Structural Mechanics, Faculty of Civil Engineering and Architecture, Lublin University of Technology, Nadbystrzycka 40, 20-618 Lublin, Poland; j.podgorski@pollub.pl

**Keywords:** X-FEM, fracture mechanics, Abaqus user subroutine, UDMGINI

## Abstract

This paper describes a method of predicting the direction of crack propagation implemented by user subroutines in the Simulia-Abaqus FEA system with the use of the extended finite element method (X-FEM). This method is based on displacements and stresses according to Westergaard’s solution of Griffith’s crack problem. During the calculations, in each crack increment, the algorithm reads the stresses and displacements in the model around the crack tip, calculates the criterion values at the read points, reduces them to a unit distance from the crack tip, fits a polynomial to these points, and finds the minimum of the function closest to the last propagation angle. The algorithm also decides when the crack grows, depending on a chosen criterion. Four criteria have been implemented to predict the direction of failure propagation: the maximum principal stress criterion, the Ottosen–Podgórski criterion, the new criterion described here based on the minimum component values of the displacement vector, and the maximum circumferential tensile stress (MTS). These criteria were verified in two tests: the three-point bending test of the notched beam and the anchor pull-out test. For these tests, the criterion built into Simulia Abaqus does not correctly define the crack path, which causes the crack propagation direction to “rotate” when simulating the fracture. The criteria developed here, in most cases, determine the crack path and the maximum force very well compared to real laboratory tests.

## 1. Introduction

This manuscript presents the results of fracture simulation with the use of a novel implementation of the crack propagation criteria with the user subroutine in the Simulia-Abaqus FEA system [1]. Four failure criteria were implemented: the maximum principal stress criterion, the Ottosen–Podgórski criterion, the maximum circumferential tensile stress (MTS) criterion, and a novel criterion based on displacements around the crack tip. Fractures were simulated using the extended finite element method (X-FEM), which is the most popular numerical technique based on the generalized finite element (GFEM) and the partition of unity (PUM) methods used to solve discontinuous mechanical problems, especially to simulate crack growth [2]. The geometry of the elements that are subject to fracture is supplemented with the enrichment function (Heaviside function), i.e., the jump function that is responsible for disconnecting a single finite element. The crack path is thus, in theory, independent of the mesh of elements.

An important limitation related to the X-FEM method is the lack of direct stress values at the crack tip, but only for the surrounding integration points. Most often, commercial FEM systems adopt very simple assumptions in determining the direction of crack propagation. The Abaqus system analyzes the stresses at single integration points in a finite element, regardless of the stresses around that point. However, the system provides tools that allow the user to improve these methods. The user subroutine to define the damage initiation criterion (UDMGINI) is responsible for the implementation of its own material failure criterion, which uses selected input data to calculate the material effort value at the crack tip and the direction of the crack propagation in each load increment in the simulation. This work describes a novel crack simulation method and includes the results of computer simulations of cracking performed with and without this method. To verify the operation of the subroutines, the three-point bending test of the notched beam and the anchor pull-out test were analyzed. The proposed method copes well with simulating brittle fracture.

In the literature, few works describe crack simulation using the UDMGINI subroutine in the Abaqus system. In Zuo et al., concerning brittle materials [3] where the authors’ own subroutine was used, the influence of the arrangement of sedimentary rock layers on the fracture propagation angle was investigated. Studies have been conducted on the fatigue of the surface layer of the material under the influence of cyclic friction [4,5]. In most other studies, the cracking of steel [6,7], composites [8,9], or polymers [10] has been investigated. However, no work focused on the fracture of brittle materials. In addition, none of the above works focused on the effective improvement of the Abaqus method of simulating crack propagation using X-FEM.

## 2. Numerical Subroutine for Predicting the Direction of Crack Propagation

A subroutine is a program code written in Fortran that can replace a fragment of the Abaqus solver algorithm. In this work, it was necessary to perform three different subroutines, each with a different task. The implementation of these subroutines enabled the use of any failure criterion.

To explain how the subroutines work, we start by explaining the algorithm of the Abaqus system during the calculations. Calculations in a solver that uses Abaqus are divided into individual stages. The largest stage is the step, which is the stage of the calculation given by the user. The steps are divided into increments. They allow for a smooth linear load increase so that at the post-processing stage, it is possible to read the results appearing during the model loading. The increments are divided into iterations. In each iteration, displacements at all integration points in all finite elements of the model are calculated. The X-FEM in Abaqus allows for the use of only four-node elements; however, it is possible to choose between a regular four-node plane stress element with four integration points (CPS4) and the same element with reduced integration, i.e., with one integration point (CPS4R). It was decided that the created subroutines would only work with the CPS4 elements, because four times as many data points will produce more accurate results. Similarly, for an axially symmetric task, 4-node axisymmetric elements (CAX4) are used.

**Figure 1:**
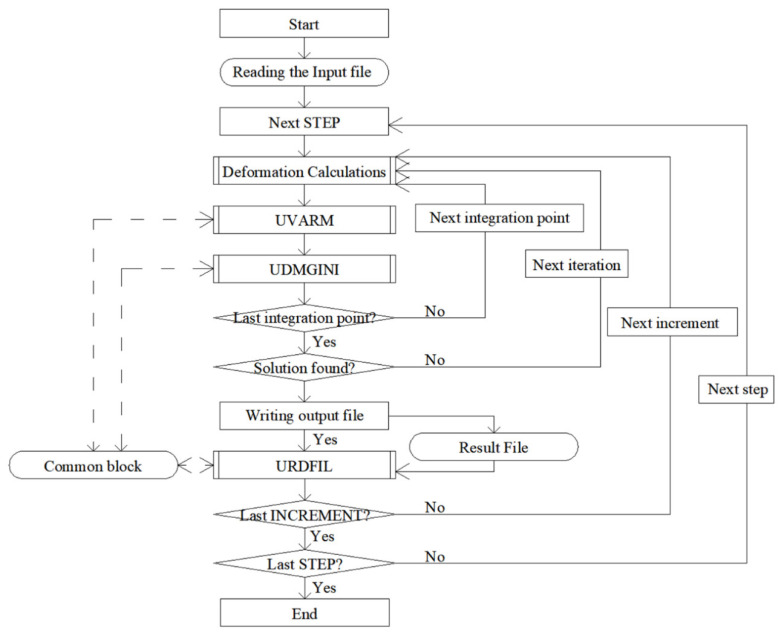
Simplified Abaqus finite element method (FEM) algorithm containing the necessary subroutines. Here, UVARM is the user subroutine to generate element output, UDMGINI is the user subroutine to define the damage initiation criterion and URDFIL is the user subroutine to read the results file.

As shown in the algorithm in Figure 1 the program starts the user subroutine to generate element output (UVARM) subroutine and then the UDMGINI subroutine after each calculation of displacement at each integration point. Before each increment, Abaqus saves the selected results to the results file (a selection of the data is saved in the appropriate place in the input file by the user). The data are then read from the results file by the user subroutine to read the results file (URDFIL) subroutine. The UVARM and UDMGINI subroutines are launched many times more than the URDFIL subroutine. Theoretically, the whole process could be performed without the URDFIL subroutine, but the computation would take incomparably longer. It was decided that the most important calculations would be performed in the URDFIL subroutine to reduce the simulation time. Additionally, there is a place in the computer’s memory called the common block. It is a memory common to all subroutines, and it allows the results of calculations to be transferred between subroutines.

UVARM is used to read the results available for the currently analyzed integration point. The UDMGINI subroutine is used to define the direction of the crack propagation and the value of the material effort. The URDFIL subroutine is responsible for reading the results file before each load increment and using the common block for transmitting these results to other subroutines. In this case, it was also used to calculate the crack propagation angle *α* from the read results.

Abaqus works very simply in the default method of determining the direction of fracture propagation using the criterion of maximum principal stresses, without the use of subroutines. It reads the stresses at each integration point that belongs to an element with an enrichment function. These are all elements at the beginning of the simulation, and after the fracture is formed, the three elements closest to the crack tip are included. Then, these stresses are rotated to the principal stresses. The rotation angle of these stresses is the angle of the fracture propagation for its next increment. A crack occurs when the material effort is greater than 1. Material effort in this case is the maximum principal stresses found in the model divided by the user-specified tensile strength value. Unfortunately, in the case of a four-node element, Abaqus averages the value of the four integration points in one element. Most often, the stresses at one integration point exceed the tensile strength, but the average does not yet, which is a misconception. Additionally, Abaqus averages the four rotation vectors of the stress tensor, which also means that the direction of propagation is often incorrect. The tensor rotation is significantly influenced by shear stresses, which are often disturbed near the crack tip. The method described below is a solution to all the problems mentioned above.

When the UDMGINI subroutine is performed for the first three integration points in an element, the program only collects data and remembers them until it reaches the last integration point in the element. For the first three elements, the criterion value and the crack propagation direction vector are defined as 0. As Abaqus averages the values of the crack propagation direction vector from four integration points, it was found that the average of one determined vector for the fourth integration point and three zero vectors will provide a vector directed in the same direction. Similarly, for the maximum principal stress criterion, the algorithm finds the maximum principal stresses at the four integration points, and while processing the fourth point, it selects the largest value of the four (the integration point with the highest effort) and then multiplies it by 4, so that the program (when calculating the arithmetic mean) obtains the real value closest to the crack tip. The crack propagation direction vector for this method is counted in the URDFIL subroutine and only the final value is passed to the UDMGINI subroutine.

**Figure 2:**
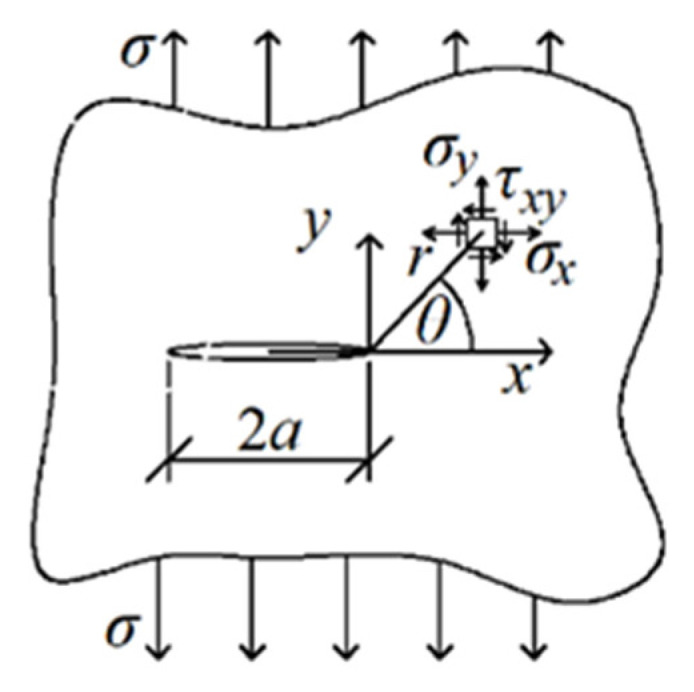
Griffith’s crack.

We assumed that around the hypothetical crack in any model, the stress field would be close to the Westergaard solution for the Griffith fracture (Figure 2). This task is symmetrical along the initial crack, which means that the crack should propagate at an angle of 0°. The stresses around the crack tip in the polar coordinate system for Mode I of loading are as follows (assuming the stress intensity factor = 1):

(1) $$\sigma_{x}\left(r,\theta \right)=\frac{1}{\sqrt{r}}\cdot \cos\left(\frac{\theta }{2}\right)\cdot \left[1-\sin\left(\frac{\theta }{2}\right)\cdot \sin\left(\frac{3\cdot \theta }{2}\right)\right],\;\sigma_{y}\left(r,\theta \right)=\frac{1}{\sqrt{r}}\cdot \cos\left(\frac{\theta }{2}\right)\cdot \left[1+\sin\left(\frac{\theta }{2}\right)\cdot \sin\left(\frac{3\cdot \theta }{2}\right)\right],\;\tau_{xy}\left(r,\theta \right)=\frac{1}{\sqrt{r}}\cdot \sin\left(\frac{\theta }{2}\right)\cdot \cos\left(\frac{\theta }{2}\right)\cdot \cos\left(\frac{3\cdot \theta }{2}\right).$$

**Figure 3:**
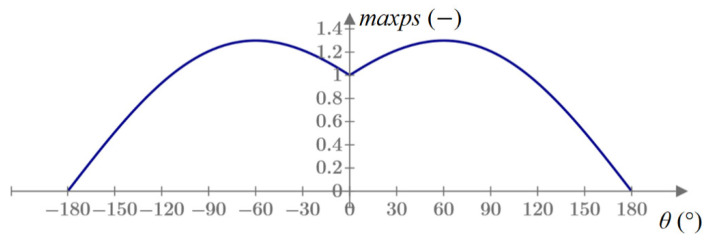
Maximum principal stresses around Griffith’s crack in Mode I.

By substituting the radius *r* of any value (e.g., 1) and converting the above formulas to the principal stresses, for an angle of 0°, there is a local minimum value of the principal stresses in a certain range of angles (adopted from −60° to 60°), as shown in Figure 3.

The absolute conditions that must be met by the angle of the crack propagation were determined:

- It must be zero for the first derivative of the function (because this point is the extreme),
- The second derivative of the function must be positive (because it is the minimum), and
- This is the value closest to the angle found in the previous load increment (since sometimes two angle values meet the above two conditions).

**Figure 4:**
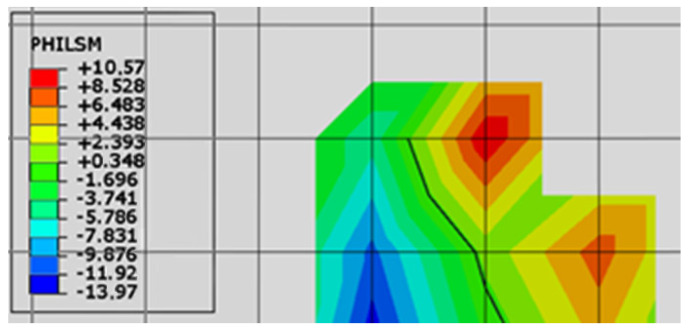
Explanation of the values of *φ* function of the level set method (PHILSM).

The URDFIL subroutine first reads the following data from the results file: node coordinates, node displacements, integration point coordinates, integration point stresses, and the values of *φ* function of the level set method (PHILSM). In Abaqus, it is not possible to read the crack tip coordinates directly from the results file. Therefore, before the operation of the crack propagation algorithm will be presented, the data from Abaqus, named PHILSM, should be explained. PHILSM is a result that occurs only on cracked finite elements. On one side of the crack, it has positive values; on the other, it has negative values. With a linear fit, the crack is where the PHILSM takes the value of 0 (Figure 4). Knowing the PHILSM values in the nodes and their coordinates, the algorithm determines the coordinates of the crack tip.

Based on the figure above, we found that the crack tip localized in an element that had one node with a negative PHILSM value, one node with a positive PHILSM value, and two nodes without a PHILSM value. Based on the linear interpolation of the coordinates of the nodes with PHILSM values, the coordinates of the crack tip are searched.

Based on changes in the PHILSM value, the algorithm also checks whether a crack is already present in the model and whether there has been an increase in the crack propagation in the analyzed increment. The program searches the direction of the crack propagation only in increments when in the previous increment there was an increase in the crack length. This saves computation time.

After finding the crack tip coordinates, the origin of the coordinate system is transferred to the crack tip, and the angle of the last crack propagation is set to 0°. Next, the algorithm searches for several dozen integration points around the crack tip; it reads the stresses at these points and then counts the values of the maximum principal stresses at these points. There should be enough points (no less than 30 were set) to obtain an accurate graph of a function similar to the Westergaard function. There must not be too many points (no more than 300 were set) so that points too far from the crack tip are not considered and so that the other stress concentration points in the model do not interfere with the results.

In the next step, these stresses are reduced to a unit radius, as shown in Figure 3. Following Equation (1), for this purpose, the stresses are multiplied by $\sqrt{r}$, where *r* is the distance of the crack tip from the analyzed integration point.

Based on these values, it is possible to create a plot of the maximum principal stress versus the angle around the crack tip, which should be similar to the plot in Figure 3. The sixth-degree polynomial is fitted to these points using the least-squares method. Then, a point that meets three conditions is searched, i.e., the zero of the derivative of this polynomial is searched by the bisection method (the closest to the angle of the crack propagation from the previous increment). The value obtained is the next crack propagation angle, which is passed to the UDMGINI subroutine.

**Figure 5:**
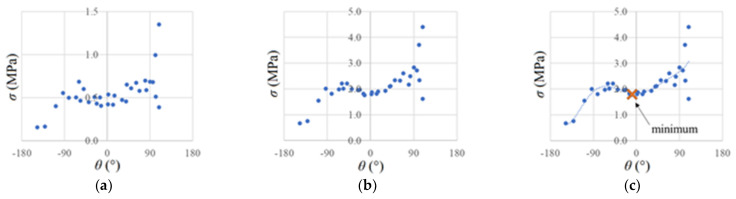
Calculating the new crack angle around the crack tip: (**a**) principal stresses; (**b**) principal stresses reduced to a radius = 1; (**c**) the 6th-degree polynomial is fitted and the minimum is found.

The operation of this algorithm is shown in Figure 5. In Figure 5a, the values of the principal stresses are inserted around the crack tip in an exemplary calculation step. In Figure 5b, these stresses are divided by $\sqrt{r}$, and in Figure 5c, the polynomial is fitted and the minimum is found near the previous crack propagation angle (0°). This angle is the new crack propagation angle *α*, which is transferred to the UDMGINI subroutine.

None of the above steps can work when the crack has not yet appeared in the model. The difficulty in locating the initial fracture is a known problem in the X-FEM. In such a case, it was decided that the coordinates of the crack tip are taken as the coordinates of the integration point where the highest maximum principal stresses occur, while the direction of the crack propagation from the previous increment was assumed as the direction from the outside of the model to the inside, so this can help to find the first crack propagation direction in the simulation.

The criterion of the minimum gradient of the effort function for the Ottosen–Podgórski (O–P) failure criterion [11,12] was implemented very similarly. The O–P failure criterion is expressed as a relationship of three alternative stress tensor invariants:

(2) $$\sigma_{0}-C_{0}+C_{1}P\left(J\right)\tau_{0}+C_{2}\tau_{0}^{2}=0,$$

where *P*(*J*) is a function describing the cross-section of the boundary surface by a deviator plane *σ*_0_ = const., and is given by the equation $P\left(J\right)=\cos\left(\frac{1}{3}\arccos\left(\alpha J\right)-\beta \right)$; *α*, *β*, *C*_0_, *C*_1_, and *C*_2_ are material constants that can be obtained from the formulas presented in [12,13].

In this case, instead of the maximum principal stresses around the crack tip, the values of the material effort function *μ* is calculated as:

(3) $$\mu \left(r,\theta \right)=\frac{\sqrt{\sigma_{0}^{2}+\tau_{0}^{2}}}{\sqrt{\sigma_{f}^{2}+\tau_{f}^{2}}},$$

where *σ*_0_ and *τ*_0_ are the normal and tangential octahedral stresses determined at the tested point around the crack tip in the polar system, respectively; and *σ_f_* and *τ_f_* are the values of critical stresses proportional to *σ*_0_ and *τ*_0_, respectively [13].

**Figure 6:**
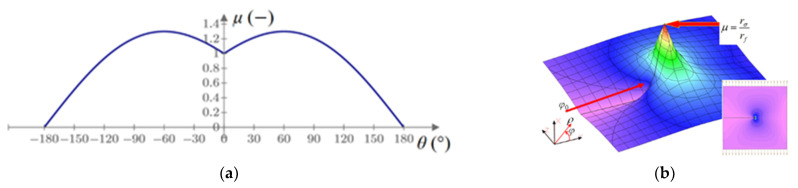
The material effort *μ* around the crack tip: (**a**) graph of *μ* for a unit radius; (**b**) three-dimensional graph of *μ* around the crack tip.

By inserting the Westergaard’s stresses as data around the crack tip, the material effort *μ* also takes a local minimum in the direction of the crack propagation, which can be seen in Figure 6.

The algorithm differs in two places from the criterion of the maximum principal stresses. The calculation of the crack propagation angle in the URDFIL subroutine is calculated using the material effort instead of the principal stresses. In the UDMGINI subroutine, the decision of crack initiation is made when the material effort is greater than 1.

The novel criterion, called the minimum displacement criterion, was created next. It is based on the distribution of displacements around Griffith’s crack, which in Mode I of the crack loading is described by the following formulas:

(4) $$u_{x}=\sqrt{r}\left[\left(\kappa -1\right)\cos\left(\frac{\theta }{2}\right)+\sin\left(\theta \right)\sin\left(\frac{\theta }{2}\right)\right],\;\;u_{y}=\sqrt{r}\left[\left(\kappa +1\right)\sin\left(\frac{\theta }{2}\right)-\sin\left(\theta \right)\cos\left(\frac{\theta }{2}\right)\right],\;\kappa =\left\{\begin{array}{c} 3-4\nu \;\mathrm{for plane strain}\\ \frac{\left(3\;-\;\nu \right)}{1\;+\;\nu }\;\mathrm{for plane stress} \end{array}.\right.$$

**Figure 7:**
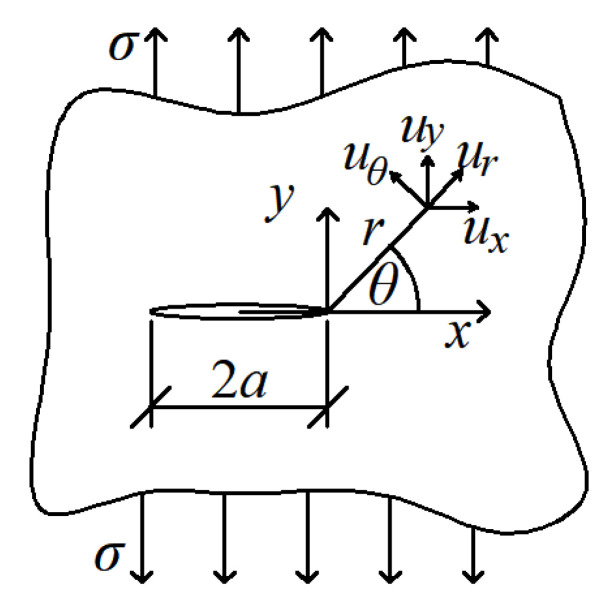
Displacements around Griffith’s crack.

**Figure 8:**
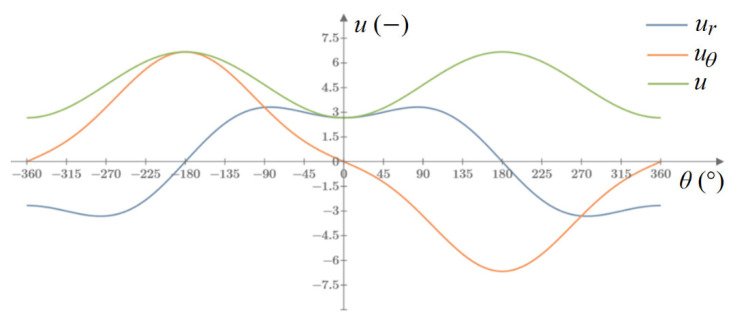
A plot of displacements around the tip of Griffith’s crack for any radius.

*u_x_* and *u_y_* are horizontal and vertical displacements at any point in the polar system around the crack tip, respectively, assuming that the crack tip does not move (e.g., due to the movement of the entire model under load). The diagram of these displacements is shown in Figure 7. After rotating these displacements by angle *θ* into displacements *u_r_* and *u_θ_*, in the crack propagation direction:

- Displacements along the radius *u_r_* take the minimum value;
- Displacements perpendicular to the radius *u_θ_* = 0 or *d*^2^*u*/*dθ* ^2^ = 0 (min *du_θ_*);
- The resultant displacements *u* have a minimum value;
- We decided to use the third condition. This situation is illustrated in Figure 8.

The difference between the subroutines is that the stresses at the integration points are not read, but the displacements at the nodes are. In the URDFIL subroutine, the resultant displacements are read for at least 30 points around the crack tip, then the displacements are shifted so that at the crack tip, as in the Westergaard’s formula, the displacements are zero (because the model usually moves during the simulation). The displacements are reduced to a unit radius according to the relationship described in Equation (4). A 6th-order polynomial is fitted and the minimum is found.

Unfortunately, using this criterion, it is not possible to determine the crack initiation condition. Thus, the criterion of maximum principal stresses was used in the UDMGINI subroutine. Only the direction of the crack is defined by the displacements.

The last criterion modeled is the maximum circumferential tensile stress (MTS) criterion [14]. It is a criterion based on stress intensity factors. In this case, the fracture occurs in the direction for which the graph described by the formula below reaches the local minimum:

(5) $$\sigma_{\theta \theta }\left(r,\theta \right)=\frac{K_{I}}{\sqrt{2\pi r}}\left(\frac{3}{4}\cos\frac{\theta }{2}+\frac{1}{4}\cos\frac{3\theta }{2}\right)+\frac{K_{\mathrm{II}}}{\sqrt{2\pi r}}\left(-\frac{3}{4}\sin\frac{\theta }{2}-\frac{3}{4}\sin\frac{3\theta }{2}\right).$$

The formula shows that as the values of the stress intensity factors *K*_I_ and *K*_II_ for the crack are known, it is not necessary to read any further data from the model. We attempted to find the stress intensity factors using the direct method:

(6) $$K_{I}=\frac{2\mu u_{y}}{\kappa +1}\sqrt{\frac{2\pi }{r\to 0}},\;K_{\mathrm{II}}=\frac{2\mu u_{x}}{\kappa +1}\sqrt{\frac{2\pi }{r\to 0}}.$$

**Figure 9:**
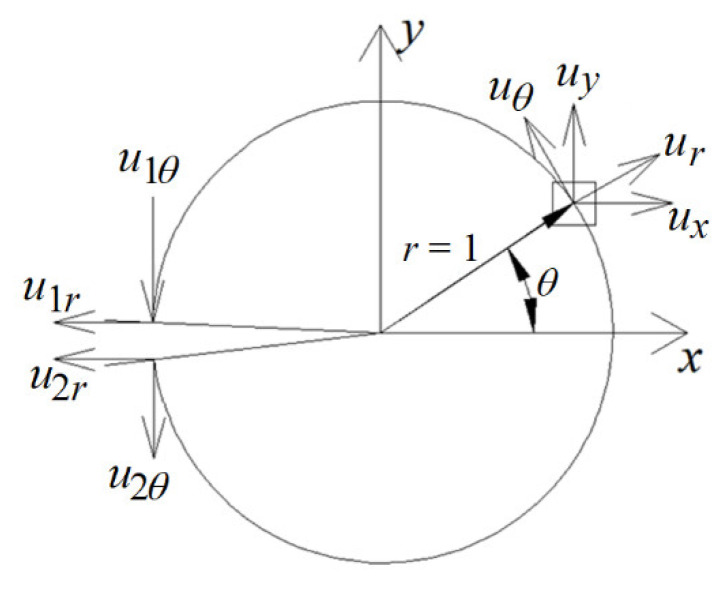
Diagram of determining the displacements on both sides of the crack.

To find the direction of the crack propagation, it is not necessary to find the exact values of the coefficients *K*_I_ and *K*_II_, only the relations between them. For this reason, the constant values and the influence of the distance from the crack tip can be removed. Hence, the values of the coefficients take a simple form:

(7) $$\frac{K_{I}}{K_{\mathrm{II}}}=\frac{u_{y}}{u_{x}},$$

where *u_x_* and *u_y_* are the differences in displacements on both sides of the crack along the crack and perpendicular to the crack, respectively. Thus, in Figure 9, they are $u_{1r}-u_{2r}$ and $u_{1\theta }-u_{2\theta }$, respectively.

The values for *u*_1*θ*_, *u*_2*θ*_, *u*_1*r*_, and *u*_2*r*_ are found from the displacement plots *u_r_* and *u_θ_* by reading these values at the nodes around the crack tip during the simulation. Polynomials are fitted to the displacement plots, and the values for the angles of 180° and −180° are read. Unfortunately, it is not possible to accurately fit a polynomial from an angle of 0° in most cases. The values of displacements very close to the crack calculated during the simulation are often inaccurate and distorted by the crack. For this reason, as will be shown in the next section, the obtained values of *K*_I_ and *K*_II_ are far from reality; therefore, the direction of the crack propagation is less precise than for the previous criteria.

Since the *K*_I_ and *K*_II_ values are calculated with the unit values of *μ* and *κ* in Equation (6), this criterion cannot be used to determine crack initiation in the UDMGINI subroutine. Again, in this case, the criterion of maximum principal stresses was used.

**Figure 10:**
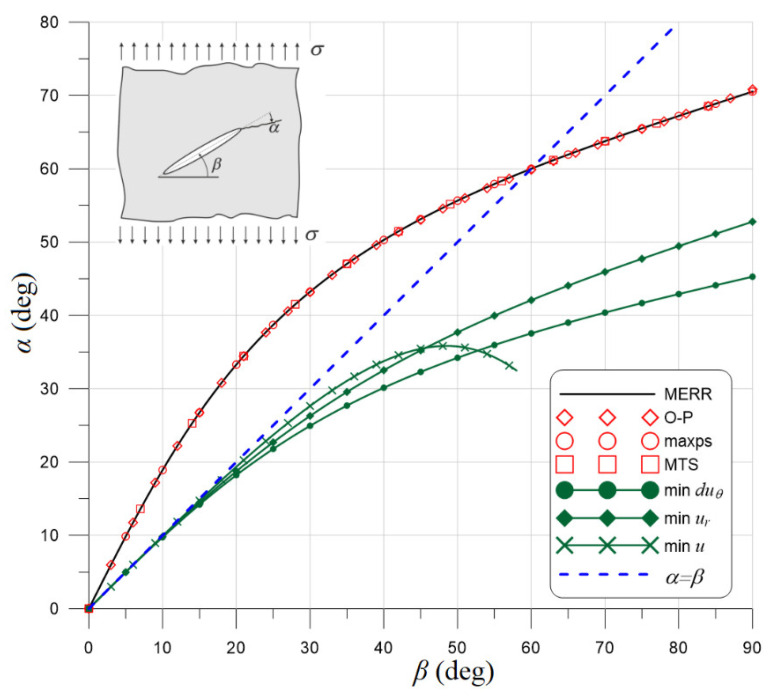
Comparison of the implemented criteria predicting the direction of the crack propagation with the maximum energy release rate (MERR) criterion. O–P: minimum gradient of the effort function for the Ottosen–Podgórski failure criterion, maxps: maximum principal stress, MTS: maximum circumferential tensile stress, displacements criteria: (min *du_θ_*, min *u_r_*, min *u*), *α=β*: direction normal to applied stress.

The comparison of the applied criteria predicting the direction of the crack propagation with the maximum energy release rate (MERR) criterion [15,16] in the case of unidirectional tensile state is shown in Figure 10. As can be seen, the criteria based on stresses give practically identical values of the propagation angle in this case. The differences between these criteria become apparent only in the case of two-directional stress states. The criteria based on the analysis of displacements around the crack tip give results that are significantly different from the stress criteria, which becomes very visible for larger slopes of the crack.

## 3. Laboratory and “In Situ” Tests to Verify the Implemented Subroutine

In order to verify the correctness of the implemented method of predicting the crack propagation path, two laboratory tests were simulated: three-point bending of a beam with a notch and an anchor pull-out test. These results were compared with the results of tests carried out in the laboratory and under “in situ” conditions.

The adopted material was sandstone, and the material parameters of which were taken from previous author’s work [17]:

- Compressive strength *f_c_* = 92.563 MPa,
- Tensile strength *f_t_* = 3.069 MPa,
- Young’s modulus *E* = 13.727 GPa,
- Poisson’s ratio *ν* = 0.148,
- Critical strain energy release rate in mode I *G*_I*C*_ = 47.946 N/m.

The above results were obtained on the basis of our own laboratory tests. The average compressive strength was obtained from the uniaxial compression test of 12 cubic samples. Before the samples were destroyed, the Young’s modulus and Poisson’s ratio were determined from the compression test of samples with four strain gauges glued in the horizontal and vertical directions on the edges of the sample. The tensile strength was obtained from the three-point bending test of four beams and the "Brazilian" test of 9 cubic samples. Critical fracture energy *G*_I*C*_ in the I mode was determined on the basis of a three-point bending test of three notched beams. The energy value was determined using the method of Brown and Srawley formula [18].

The description of computer simulations without and with the use of the novel proprietary method of crack propagation with a comparison of the implemented failure criteria are presented below. The failure criterion selected for simulation without the novel method is one of the default criteria recommended in the Abaqus documentation, the maximum principal stress (MAXPS) criterion. As previously described, this criterion rotates the stresses at the integration points into principal stresses and leads the crack according to the rotation vector of the stress tensor when the maximum stresses exceed the tensile strength.

**Figure 11:**
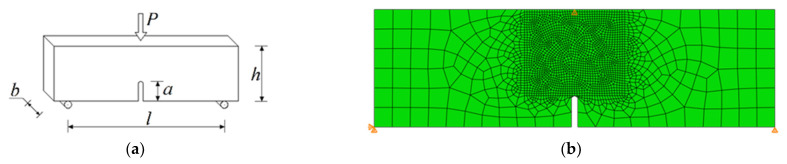
Test of three-point bending of a beam with a notch: (**a**) task scheme; (**b**) computer model mesh.

The first test chosen was a three-point beam bending with a notch. The test scheme is shown in Figure 11a. This test was ideal to carry out first because the fracture line is very predictable, even without simulation. The task is axially symmetrical, so the fracture will certainly run vertically from the cut to the point where the load is applied.

The dimensions of the sample were assumed to be the same as for one of the beams in the laboratory test described in [17], i.e., *h* = 93.7 mm, *b* = 90.22 mm, *l* = 320 mm, and *a* = 25 mm. The model was constructed in a plane stress state. The mesh size varied from 2 to 15 mm, and the mesh is presented in Figure 11b. To reduce the dependence of the crack path on the mesh, it was generated chaotically in the area of the planned crack path.

**Figure 12:**
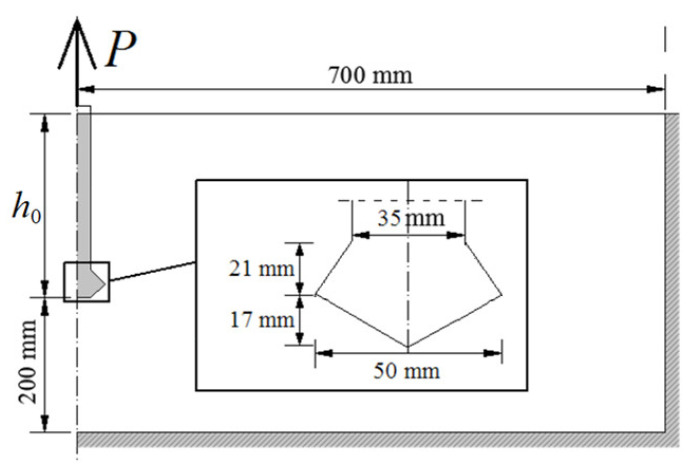
Self-undercut anchor: (**a**) before installation; (**b**) after use.

Another test for which simulations were performed was the anchor pull-out test. An attempt to pull out a self-undercutting anchor embedded in sandstone was simulated. In the analyzed study, an anchor was used that was blocked by the wings at the end of the anchor and not by the side surface. The crack simulation in this study has been extensively described in the literature [19,20]. Figure 12a shows the anchor prepared for mounting, and Figure 12b shows a used anchor with folded wings.

**Figure 13:**
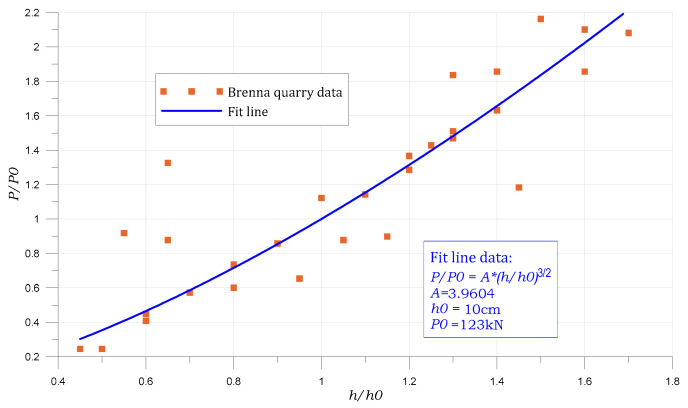
Results of tests for pulling out the anchor fixed in the rock mass of the “Brenna” sandstone. Relationship between maximum force and anchorage depth with exponential fit line.

To compare the results, a series of in situ tests of pulling out an anchor embedded in a sandstone block were performed. 31 tests were performed for different anchorage depths. The diagram of the relationship between the maximum force and the anchorage depth is presented in Figure 13. The results of these studies come from the work carried out by the teams of the Institute of Mining Technology in Gliwice and the Lublin University of Technology as part of the project financed by the Polish National Science Center: RODEST No. 2015/19 / B / ST10 / 02817 [21].

**Figure 14:**
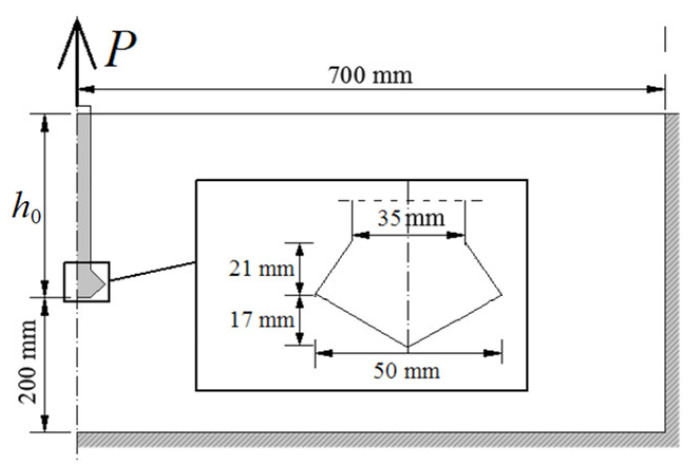
The scheme of simulating the pull-out test.

It is a 2D axially symmetric test, the diagram of which is shown in Figure 14. A large reserve of the model dimensions was assumed so that the boundary conditions did not have a large impact on the result.

**Figure 15:**
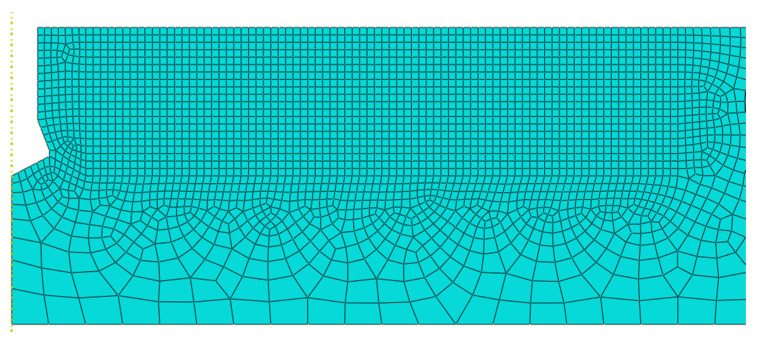
The grid of the simulation model of the pull-out test.

In Figure 14, *P* is the pulling-out force and *h*_0_ is the anchorage depth. In the computer simulation, the load on the material is transferred through contact at the undercut. In this case, the same material used in the previous study was adopted. The material of the anchor was steel, and the friction coefficient between the materials was 0.1. So far, many simulations with different anchorage depths, material parameters, different mesh, etc., have been performed. For this study, the size of the finite element mesh was from 5 to 25 mm, as shown in Figure 15. An anchorage depth of 10 cm was assumed.

## 4. Results

### 4.1. Simulation of a Three-Point Bending Beam with a Notch

**Figure 16:**
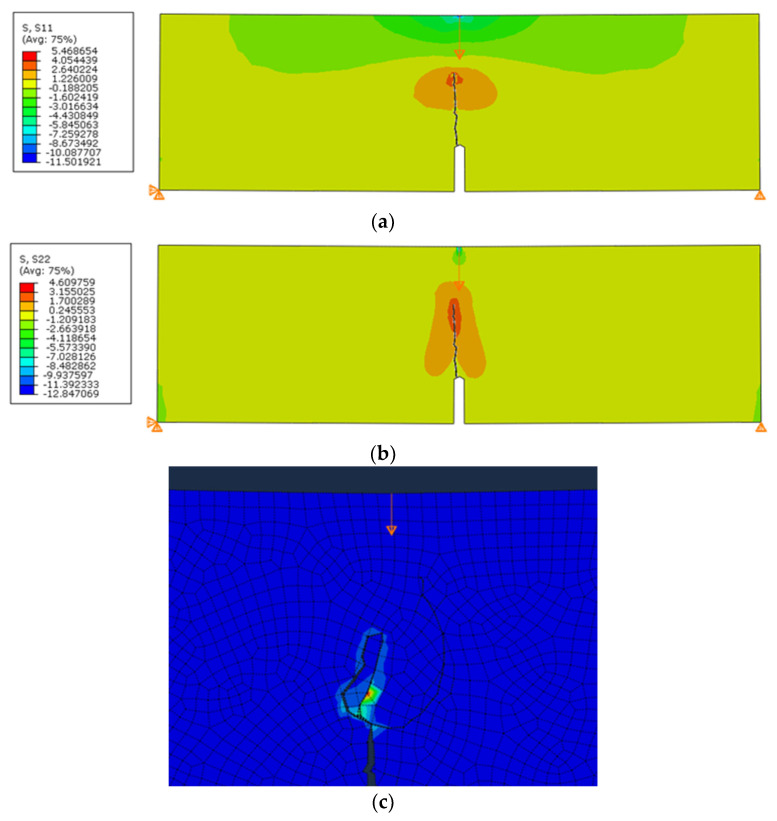
Simulation results for a three-point bending of a notched beam: (**a**) *σ*_11_; (**b**) *σ*_22_; (**c**) crack curling effect.

The above-described model of a three-point bent notched beam was simulated using the default method of predicting the direction of the crack propagation in the Abaqus system. Figure 16 shows the path obtained from this simulation. Up to a certain point, the path ran correctly, but near half of its length it began to bend in the wrong direction. The crack curled unrealistically, as shown in Figure 16c. For this reason, this result cannot be accepted.

During the test, a serious drawback of the described method was noticed. Since the crack angle is calculated individually for each finite element, it changes significantly with its individual growth. During the calculations, the angle of the crack *α* is defined by which angle the crack should be directed, and the vector is determined from it. However, due to the properties of trigonometric functions, the Abaqus program receives information that indicates that it can be an angle α, but also an angle α + 180°. Thus, the program runs a fracture only based on a feature of the X-FEM method, i.e., the fracture cannot be traced again through the same finite element. For this reason, in most cases, the fracture is guided at the correct angle α, but often the situation occurs where the designated angle α causes the fracture to pass through an already cracked finite element, and then the fracture turns back 180°.

Fortunately, the maximum force in this simulation was obtained before the fracture began to distort and was 3.433 kN. In the laboratory test for which this simulation was performed, the force was 4.211 kN.

The same simulation was performed using the novel method with the use of Abaqus user subroutines with different failure criteria.

**Figure 17:**
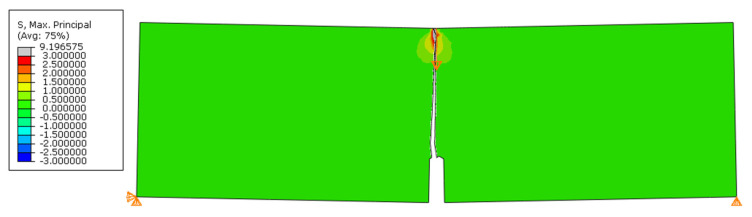
Crack path of a three-point bending beam with a notch using the novel criterion of maximum principal stresses.

As shown in Figure 17, the crack path is very realistic for the simplest criterion of the maximum principal stresses. It is smoother than from the default method and, most importantly, the crack reaches the end of the beam, as in the laboratory test. There are two reasons for this improvement. The default method considers the stresses at one integration point, and the novel method considers several dozen integration points around the crack tip. The shear stresses *τ*_12_, which are often disturbed in the area of the crack tip, have a strong influence on the angle of rotation in the default method. In the novel method, shear stresses are not so important. Stresses at a certain distance from the crack tip, in which there are no disturbances caused by defects in the finite element method, have a strong influence on the correct results. The maximum force obtained in the calculations was 3.167 kN, which is still far from the laboratory test result, probably due to the large dispersion of the test results, which indicated the heterogeneity of the sandstone used. In the future, therefore, tests and simulations should be conducted on a more homogeneous material, such as concrete. The maximum force was lower than for the built-in method because the stresses were obtained from the most strenuous integration point and not averaged over four points in one element.

**Figure 18:**
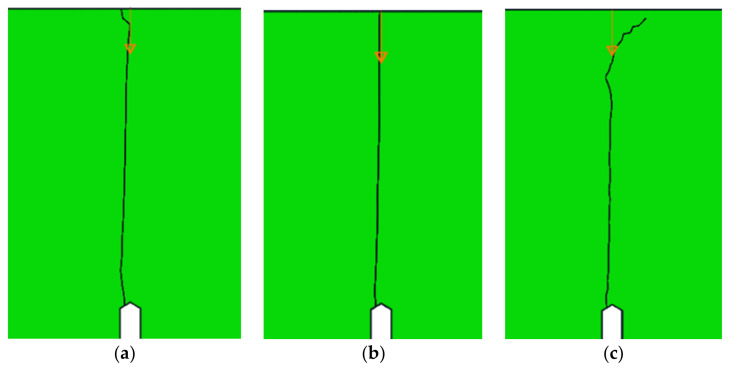
Crack paths of a three-point bending beam in the remaining criteria: (**a**) the Ottosen–Podgórski criterion; (**b**) the minimum displacement criterion; (**c**) the maximum circumferential tensile stress (MTS) criterion.

The results obtained for the other criteria were also satisfactory. The fracture line obtained for the Ottosen–Podgórski criterion (Figure 18a) is very similar to the line obtained by the maximum principal stress criterion because both criteria are based on stresses.

The fracture line obtained using the minimum displacement criterion (Figure 18b) is very straight and vertical along its entire length, which reflects the real fracture of such a beam. An accurate vertical line was obtained because the task was symmetrical, and therefore the displacement field around the crack tip was also symmetrical, so the minimum value of the resultant displacements was always obtained in the vertical direction.

The crack path obtained using the MTS criterion (Figure 18c) was the least accurate among the implementations tested, but still much closer to reality than the line calculated by the default Abaqus method. This inaccuracy probably occurred due to the non-ideal programmed method for calculating the values of the stress intensity factors.

### 4.2. Simulation of Pull-Out Test

**Figure 19:**
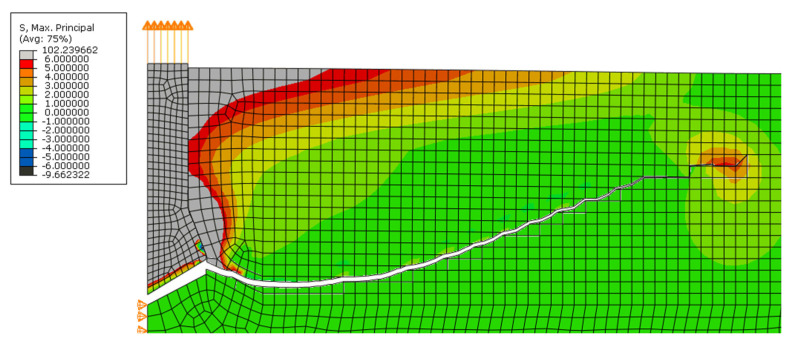
The simulation result of the pull-out test with a modeled anchor with an anchorage length of 10 cm and a friction coefficient of 0.1.

In all simulations using the built-in maximum principal stress criterion, the fracture was correct up to a certain point, and then the fracture line disturbance began, after which the calculation was interrupted or the crack turned as in the three-point beam bending test. Figure 19 shows the result of the crack path for the simulation with the parameters adopted for this study. In this case, the maximum force was obtained before the disturbance started to appear in the model. The value of the maximum force was approximately 123 kN (dependent on the size of the mesh), while in the same test performed in situ, the mean force was the same. We found that the stresses in Abaqus are correctly calculated, but there are problems with correctly finding the crack propagation direction.

**Figure 20:**
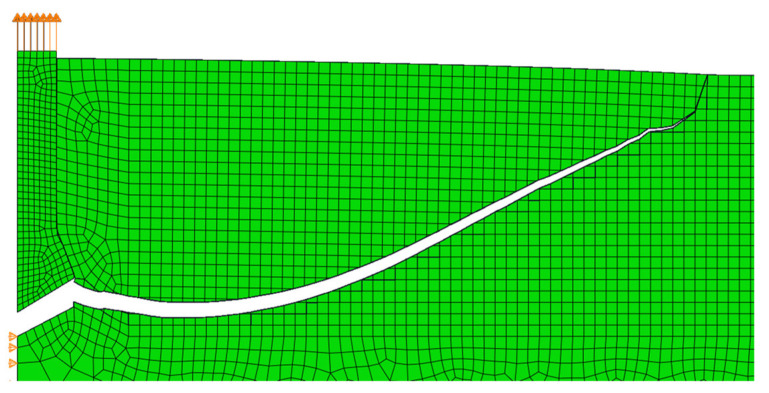
The crack path of the pull-out test using the novel implementation of the maximum principal stress criterion.

**Figure 21:**
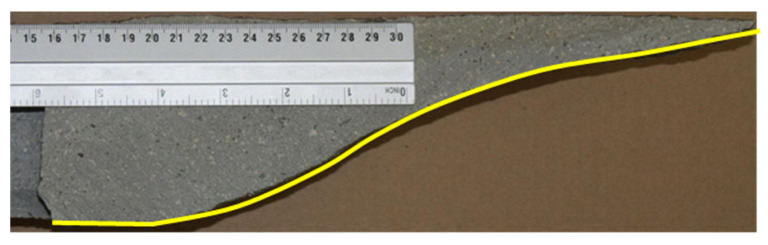
Cross-section of the pulled-out fragment in the in situ pull-out test.

**Figure 22:**
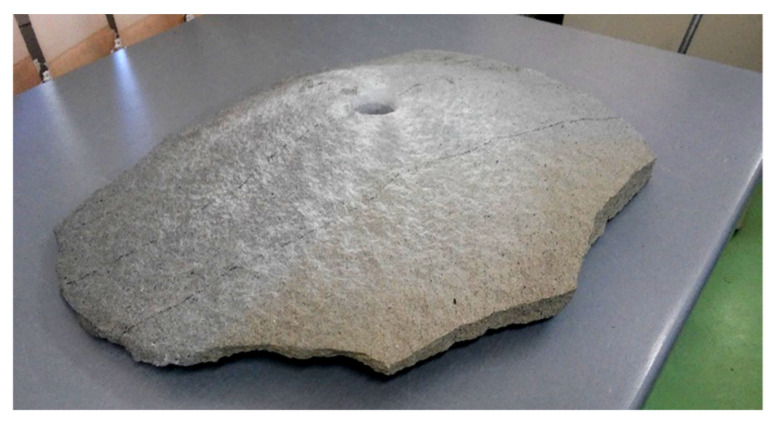
One of the elements pulled out during the pull-out test.

Then, the same test was performed using the novel method of predicting the direction of the crack propagation. The crack path obtained in the simulation using the implementation of the maximum principal stress criterion is shown in Figure 20. The maximum force was approximately 118 kN. As in the case of the previously described study, this value is lower than from the built-in method. The crack path was much closer to reality (Figure 21). The calculation did not stop and the crack did not return; it just ended. The breach in the vertical direction close to the surface of the material was also maintained. This phenomenon can be observed on the actual pulled-out element in Figure 22, where the crack suddenly twists, leading to breakage of the element. In the study on real rock, the fracture flattens before this breach, as shown in Figure 21. Unfortunately, this phenomenon was not observed in the simulation.

**Figure 23:**
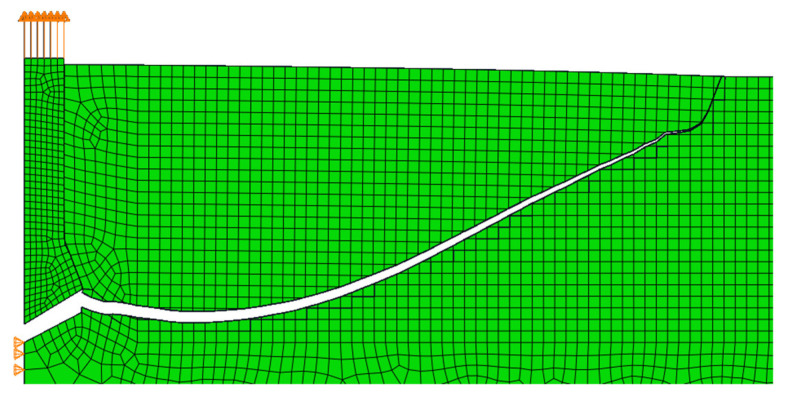
The crack path of the pull-out test using the minimum gradient of the effort function for the Ottosen–Podgórski failure criterion.

Figure 23 shows the result of the simulation using the implementation of the Ottosen–Podgórski criterion. The shape is almost identical to the previous one. The maximum force is also similar.

**Figure 24:**
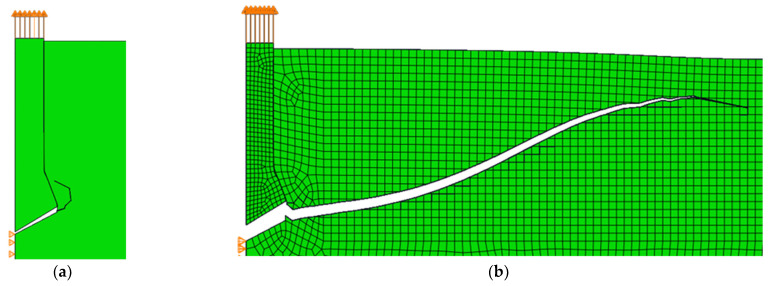
The crack path of the pull-out test using the minimum displacement criterion: (**a**) the minimum resultant displacement criterion; (**b**) the criterion of minimum displacement alongside the radius *u_r_*.

In the case of the criterion of minimal resultant displacements, the simulation was unsuccessful, as shown in Figure 24a. The nature of the axisymmetric task is most likely not appropriate for this criterion. However, we decided to modify the algorithm. Instead of the resultant displacement diagram, the displacement diagram along the radius *u_r_* was used, and the angle for which the function takes the value of the local minimum was also searched. Interestingly, for this variant, the crack path was much closer to reality (Figure 24b), but in the place where a vertical breach should appear, the fracture began to travel downwards, which is far from reality. In the future, this criterion should be examined to understand which conditions should be used to determine the crack propagation angle.

**Figure 25:**
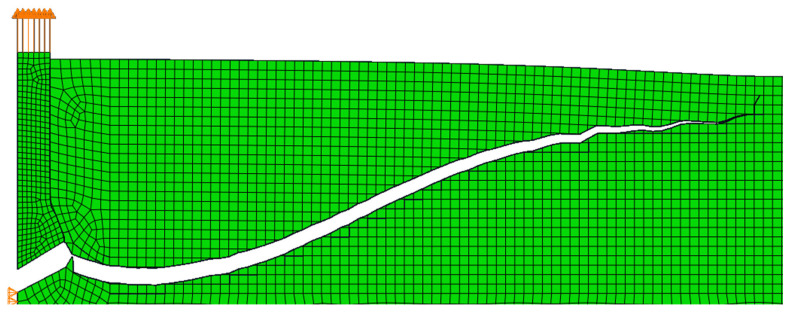
The crack path of the pull-out test using the MTS criterion.

The surprisingly correct shape of the crack path was obtained using the MTS criterion, as shown in Figure 25. The diameter of the pulled-out cone was larger than in the case of the maximum principal stress and Ottosen–Podgórski criteria, which is consistent with the in situ test. A flattening of the crack path near the surface and a vertical breakout at the end of the simulation were observed.

**Figure 26:**
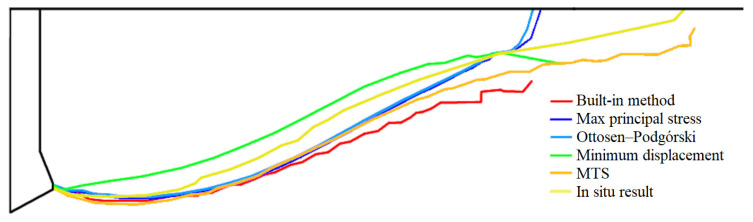
Crack paths for all methods.

Crack paths obtained from all analyzed criteria and for one in situ result are summarized in Figure 26.

## 5. Discussion

A novel method of predicting crack propagation implemented in the Abaqus FEA system produces very good results. The accuracy of the results compared to the built-in maximum principal stress criterion is improved. In most cases, the programmed failure criteria are very good at determining the crack path and the forces at which the crack propagates. The criteria that best determine the direction of crack propagation are the maximum principal stress criterion and the Ottosen–Podgórski criterion. The MTS criterion well-determined the crack path in the pull-out test, despite the simple method of finding the stress intensity factors.

An attempt to improve the algorithm is planned in the future. The criteria described also require some corrections. The MTS criterion could be improved so that not only the direction but also the force at which the crack propagates is determined by this criterion instead of the maximum principal stress criterion. The novel criterion based on displacements should also be improved by selecting the correct condition in which the direction of the crack will be determined.

Future work will involve adding more crack propagation criteria, modifying the algorithm to simulate three-dimensional models, and analyzing other experiments and materials, including non-homogeneous materials.

Unfortunately, the user, when using the UDMGINI subroutine, only has influence on defining the criterion of crack initiation and the angle of crack propagation. There is no user access to actions determining how the Abaqus system handles these data. For this reason, in the far future, the creation of a novel FEM program with the possibility of simulating cracking using the X-FEM method is proposed.

## Data Availability

Data available in a publicly accessible repository.
